# Antibiotic resistance as a tragedy of the commons: An ethical argument for a tax on antibiotic use in humans

**DOI:** 10.1111/bioe.12598

**Published:** 2019-05-20

**Authors:** Alberto Giubilini

**Affiliations:** ^1^ University of Oxford Oxford United Kingdom of Great Britain and Northern Ireland

**Keywords:** antibiotic resistance, antimicrobial resistance, drug resistance, public health ethics, taxation, tragedy of the commons

## Abstract

To the extent that antibiotic resistance (ABR) is accelerated by antibiotic consumption and that it represents a serious public health emergency, it is imperative to drastically reduce antibiotic consumption, particularly in high‐income countries. I present the problem of ABR as an instance of the collective action problem known as ‘tragedy of the commons’. I propose that there is a strong ethical justification for taxing certain uses of antibiotics, namely when antibiotics are required to treat minor and self‐limiting infections, such as respiratory tract infections, in otherwise healthy individuals. Taxation would allow a reduction in consumption (given certain behavioural economics assumptions) and/or ensure that individuals internalize or compensate for their contribution to the erosion of the common good of antibiotic effectiveness. I suggest that revenue from the tax could be used to fund conservation and innovation strategies. Taxation might be a coercive policy, especially for certain individuals, but the ethical case for coercive policies is very strong when the good to be preserved is important enough and when they force individuals to do something they have a moral obligation to do anyway. I argue that, in the case of mild and self‐limiting infections, individuals have a moral duty of easy rescue and a moral duty of fairness to make their contribution to the preservation of the common good of antibiotic effectiveness by foregoing antibiotics. I also suggest that taxing antibiotics in such cases is an all things considered ethically justified policy even if it would introduce inequalities in access to healthcare.

## 1 INTRODUCTION: THE PROBLEM OF ANTIBIOTIC RESISTANCE

Antibiotic resistance (ABR) (and indeed antimicrobial resistance (AMR) in general)1The focus of this paper will be antibiotic resistance because most of the literature and documents here discussed refer to antibiotic use and its regulation, but the same considerations can be extended to antimicrobial resistance more generally. is one of the greatest public health emergencies of our age. For instance, drug‐resistant tuberculosis is on the rise and is currently causing at least 200,000 deaths every year,2O'Neill, J. (2016). Tackling drug resistant infections globally: Final report and recommendations. *The Review on Antimicrobial Resistance* (p. 10). Retrieved from http://amr-Review.org/sites/default/files/160518_Final%20paper_with%20cover.pdf, Accessed 16 Aug, 2016. but more generally the total annual number of deaths due to drug‐resistant infections is estimated at around 700,000.3Ibid.


Antibiotic consumption contributes to the development of ABR because, by killing the non‐resistant bacteria, antibiotics confer a selective advantage to the resistant ones, which can more easily proliferate. As put by the Center for Disease Control and Prevention in the U.S., ‘the use of antibiotics is the single most important factor leading to antibiotic resistance around the world: simply using antibiotics creates resistance’.4CDC (Center for Disease Control and Prevention). About antimicrobial resistance, updated 2015, Retrieved from https://www.cdc.gov/drugresistance/about.html. Accessed 17 Aug, 2016. Because of antibiotic consumption, any individual can become a carrier of resistant bacteria that expose her to greater risks in future infections5Cars, O., Murray, M., Nordberg, O., Sivaraman, S., Lundborg, C. S., So, A. D., & Tomson, G. (2008). Meeting the challenge of antibiotic resistance. *British Medical Journal*,* 337*, 726–728. and can pass resistant bacteria on to other people. The fact that ABR is a natural reaction of bacteria to the exposure to antibiotics means that antibiotic effectiveness is, by its very nature, a relatively short‐lived phenomenon. New, effective antibiotics are constantly required to replace those that become ineffective. Unfortunately, today the pipeline of new antibiotics is running dry. There is often a lack of commercial incentives for private companies to pursue Research and Development (R&D) on new antibiotics, mainly because of the cost of research, because new antibiotics would initially have to compete with the old ones for as long as these remain effective, and because the necessary implementation of antibiotic stewardship programs would limit consumption and therefore sales.6Op. cit. note 2, p. 52. Thus, urgent measures are required not only to reduce antibiotic consumption for conservation purposes, but also to fund innovation through R&D.

We need to distinguish between two main drivers of ABR: the use and abuse of antibiotics in humans and in animals in the context intensive animal farming.7Giubilini, A., Birkl, P., Douglas, T., Savulescu, J., & Maslen, H. (2017). Taxing meat: Taking responsibility for one's contribution to antibiotic resistance. *Journal of Agricultural and Environmental Ethics*,* 30*(2), 179–198. Here, I will focus on the problem caused by human consumption. Abuse of antibiotics is a significant problem: often doctors prescribe antibiotics that are not necessary, such as in cases of viral infection;8Van der Velden, A., Duerden, M., Bell, J. Oxford, J., Altiner, A., Kozlov, R., … Essack, S. Y. (2013). Prescriber and patient responsibilities in treatment of acute respiratory tract infection – Essential for conservation of antibiotics. *Antibiotics*,* 2*, 316–327. for example, in the U.S. about 40 million people are given antibiotics for respiratory issues annually, but the antibiotics are therapeutically useful only in 27 million such cases.9Op. cit. note 2, pp. 35–36. The solution to the problem of antibiotic abuse does not raise particular ethical issues though: To put it simply doctors should not prescribe unnecessary antibiotics and should be prohibited from prescribing unnecessary antibiotics, because unnecessary antibiotics contribute to a collective cost without producing any individual benefit. More challenging, from an ethical perspective, is the problem of ABR driven by therapeutically justified antibiotic consumption, where individual interest in treating infections conflicts with the collective interest in preserving antibiotic effectiveness.

Using antibiotics more responsibly could slow down the development of resistance.10Op. cit. note 2. Unfortunately, the current global trend of antibiotic consumption is going in the exact opposite direction. Of particular concern is the increase in the consumption of last‐resort antibiotics such as carbapenem and colistin. According to a recent study, the total global increase in antibiotic consumption between 2000 and 2015 was 65%. If effective policies are not implemented, global consumption is projected to increase by 200% between 2015 and 2030.11Klein, E., van Boeckel, T., Martinez, E., & Pant, S. (2018). Global increase and geographic convergence in antibiotic consumption between 2000 and 2015. *Proceedings of the National Academy of Sciences of the United States of America*, *115*(15), E3463–E3470.


It is interesting to dissect the data just provided. On the one hand, in low‐ and middle‐income countries (LMICs) the increase in consumption rate was 77%, while the consumption rate in high‐income countries (HICs) fell by 4%. On the other hand, the consumption rate in most LMICs remains lower than the consumption rate in HICs, and indeed is too low in many countries where access to antibiotics is still difficult, and as a consequence infectious diseases proliferate.12Ibid. Thus, the increase in antibiotic consumption in LMICs might be seen as an overall positive datum, as it indicates that many more people in those countries have access to healthcare than was the case 15 or 20 years ago. However, such an increase needs to be closely monitored in order to ensure that the health benefits in terms of treatment of infections are not outweighed by the harms of ABR. At the same time, the still too‐high rates – despite the slight decrease – of antibiotic consumption in HICs do unquestionably constitute an urgent problem that needs to be addressed. Based on their data, Klein and colleagues conclude that

[r]educing antibiotic consumption rates in HICs and slowing the growth rate of consumption in LMICs is urgently needed to contain the problem of resistance, particularly given the long timescales and resources necessary for development of new antibiotics. However, there is a need to balance access to essential medications, particularly in LMICs where the burden of infectious diseases likely still outweighs the burden of resistant infections and where in many countries there is a significant unmet need for antibiotics (p. E3467).13Ibid.

The last observation by Klein does not apply to all LMICs; for instance, India is a major contributor to ABR because of its too‐high consumption of antibiotics,14Kumar, S. G., Adithan, C., Harish, B. N., Sujatha, S., Roy, G., & Malini, A. (2013). Antimicrobial resistance in India: A review. *Journal of Natural Science, Biology, and Medicine*,* 4*(2), 286–291; WHO India (2018). Combating antimicrobial resistance in India. Retrieved from http://www.searo.who.int/india/topics/antimicrobial_resistance/Combating_Antimicrobial_Resistance_in_India/en/. Accessed 19 Nov, 2018. and is severely burdened by ABR.15Kakkar, M. et al. (2017). Antibiotic resistance and its containment in India. *British Medical Journal*,* 358*, 25–30. Thus, a country like India might need measures to reduce antibiotic consumption in the same way as, if not more than, HICs do. One way to reduce consumption is through investing resources in conservation strategies that reduce the need for antibiotic prescription in the first place, especially in LMICs, such as improved sanitation, better hospital infection control, and wider use of vaccines.

However, as noted above, reducing consumption is not the only measure needed. Innovation strategies through R&D of new antibiotics are equally important. For instance, the O'Neill Review on Antimicrobial Resistance commissioned by the U.K. Government advocated for an international ‘Global Innovation Fund’ for early‐stage and non‐commercial R&D for which private companies do not have sufficient commercial incentives.16Op. cit., note 2. Reaching a good balance between conservation and innovation is difficult because investing in innovation might discourage efforts to promote conservation, and vice versa.17Laxminarayan, R. (2014). Antibiotic effectiveness: Balancing conservation and innovation. *Science*,* 345*(6202), 1299–1301.


In this paper, I will argue that ABR can be counteracted by disincentivizing antibiotic use and by requiring certain consumers of antibiotics to internalize the costs of ABR or to compensate for the cost they are contributing to. In this way, conservation and innovation strategies can be pursued simultaneously by requiring consumers, in certain cases, to contribute to the funding of conservation and innovation strategies. More precisely, as I suggest in Section 4, both conservation and innovation strategies could be funded through revenue generated by taxing *in certain cases* antibiotic consumption where disincentives are more urgently needed, given high consumption rates, and where there is more potential for generating revenue to fight ABR. This is certainly the case in many HICs. I want to leave open the question whether and how the same kind of policy here proposed could be adopted in some LMICs, such as India. In most such countries, a tax on antibiotics might have very undesirable outcomes, if people were forced by economic considerations to leave infections untreated in a context where public health is already poor and the burden of infectious disease already too high, and indeed much higher than the burden of resistance. It is actually more plausible to suppose that such countries should be at the receiving end of policies aimed at improving conservation. So I wish my taxation model to be taken as a suggestion for a policy to be implemented certainly in HICs, and perhaps worth considering for some LMICs within a more general cost–benefit assessment. In Section 5, I will suggest that the ethical justification for the form of taxation I am proposing is very strong when foregoing antibiotics comes at small cost to individuals, that is, in the case of mild and self‐limiting infections in individuals who, from a medical perspective, could endure such infections without incurring significant risks. In such situations, individuals have a basic ethical duty not to consume antibiotics, which significantly strengthens the ethical justification for a taxation policy, especially if such a policy is supplemented with the provision of some nonfinancial benefit to those who have to forego antibiotics because of the tax. Finally, in Section 6, I will suggest that policies that disincentivize antibiotic use in such circumstances are *all things considered* ethically justified, even if they entail some inequalities in access to healthcare.

Before taking these steps, it is worth saying something more about the tragedy of the commons that antibiotic consumption gives rise to (Section 2) and about the type of solutions that the tragedy of the commons typically calls for (Section 3).

## 2 ANTIBIOTIC RESISTANCE AS A TRAGEDY OF THE COMMONS

Antibiotic effectiveness is a common good, or a common pool resource. These types of goods are defined, among other things, by the fact that each individual who enjoys them contributes to their erosion.

Common goods typically give rise to the collective action problem known as ‘tragedy of the commons’. To introduce the problem, Garrett Hardin famously used the efficacious metaphor of a pasture that is available to many herdsmen.18Hardin, G. (1968). The tragedy of the commons. *Science*,* 162*, 1243–1248. Hardin asked us to imagine that every single herdsman, acting merely out of self‐interest, has their cattle overgraze the commons, so that the resource's availability is progressively eroded by many individual actions. The tragedy arises because individuals do not have any incentive to abstain from contributing to the depletion of the common pool resource: for any herdsman who uses the pasture, the individual benefit is large and the harm produced is insignificant. Therefore, from a self‐interested perspective, there do not seem to be enough reasons for any individual to abstain from grazing the commons. However, if all or many herdsmen act out of self‐interest, the cumulative harm is very large and the herdsmen themselves will be harmed in the long run.

Antibiotic consumption, including therapeutically necessary consumption, presents the same conflict between short‐term individual interests on the one side, and collective and individual long‐term interests on the other. In the case of therapeutically justified antibiotic use, as Jonny Anomaly put it, ‘the benefits of [antibiotic] use are borne by the individual, the costs are socialised, and the consequent harm is the product of many independent actions‘ (p. 754).19Anomaly, J. (2013). Collective action and individual choice: Rethinking how we regulate narcotics and antibiotics. *Journal of Medical Ethics*,* 39*, 752–756.


There are, however, two important differences between the tragedy of the commons as described by Hardin and the tragedy of the commons that arises in the case of antibiotic consumption. The first difference is that, unlike the case of cattle overgrazing the commons, in the case of ABR individuals can bear at least part of the costs of their individual actions, because they can become carriers of resistant bacteria that might render subsequent antibiotic use ineffective. The second difference is that, for the same reason, any individual contribution to ABR through antibiotic use is not necessarily and not always negligible (although it can be): there is a chance that every individual exposed to antibiotics becomes a vector of resistant bacteria that infect others.20Costelloe, C. et al. (2010). Effect of antibiotic prescribing in primary care on antibiotic resistance in individual patients: Systematic review and meta‐analysis, *British Medical Journal*,* 340*, 1–11. It is safe to say that each individual contribution is at least probabilistic.

It might be suggested that, in light of these two differences, it is not appropriate to talk of ‘tragedy of the commons’ in the case of antibiotic consumption. However, I will stick with the label ‘tragedy of the commons’ to refer to antibiotic consumption because the collective action problem generated by ABR calls for types of solutions that are the same as those typically called for by tragedies of the commons in general, namely the reduction of consumption, the internalization of the costs, or the compensation for the costs imposed or to which one contributes.

## 3 ADDRESSING ANTIBIOTIC RESISTANCE: FROM ETHICS TO POLICY

According to Hardin, the solution to the tragedy of the commons needs to be ethical in nature: it is necessary that individuals prioritize the long‐term collective interest over their short‐term individual interest. An important part of what ethics is about is the prioritization of other values over one's egoistic interests. Unfortunately, in many cases, prioritizing the collective over the individual good is an ethical choice individuals are unlikely to be willing to make without some form of coercion. As Hardin put it, solving the tragedy of the commons requires some form of ‘mutually agreed upon coercion’.21Op. cit. note 18, p. 1247. The notion of ‘coercion’ is problematic from a philosophical perspective, as it is difficult to explain precisely what coercion involves or what practices can be considered coercive.22Feinberg, J. (1989). *The moral limits of the criminal law*. New York; Oxford: Oxford University Press; Frankfurt, H. (1988 [1973]). Coercion and moral responsibility. In T. Honderich (Ed.), *The importance of what we care about, in essays on freedom of action* (pp. 65–86). London: Routledge & Kegan Paul; Held, V. (1972). *Coercion and coercive offers*. In P. J. Roland and J. W. Chapman (Eds.), Nomos XIV: Coercion (pp. 49–62). Chicago, IL: Aldine‐Atherton, Inc.; Nozick, R. (1969). Coercion. In S. Morgenbesser, P. Suppes, & M. White (Eds.), *Philosophy, science, and method: Essays in honor of Ernest Nagel* (pp. 440–472). New York, NY: St. Martin's Press; Wertheimer, A. (1989 [1987]). *Coercion*. Princeton, NJ: Princeton University Press. For the purposes of the present discussion, ‘coercion’ can be taken to mean the imposition of penalties or disincentives that are large enough to give an individual strong enough reasons to make a certain choice – for example, forego antibiotics – that she would otherwise not have made.

Now, a relevant question for the justification of coercive measures is the following: does a certain individual behaviour constitute an ethical requirement on individuals, independently of the existence of policies that coerce individuals into adopting that behaviour? The question is relevant because the ethical justification for a public policy that coerces individuals into doing something they have an independent ethical obligation to do anyway is stronger than the justification for a policy that coerces individuals into doing something above and beyond their call of (moral) duty.

Arguably, when implementing a coercive policy, an authority ought to be able to offer the strongest justification possible for it.23Giubilini, A. et al. (2018). Quarantine, isolation, and the duty of easy rescue in public health. *Developing World Bioethics*,* 18*(2), 182–189. Because individual moral obligations strengthen the ethical case for coercive policies, it is important to ask two distinct types of questions regarding antibiotic consumption: first, the question as to what type of coercive policies can ensure that the common good of antibiotic effectiveness is preserved as much as possible, compatibly with provision of sufficiently good health care; second, in order to assess whether these policies are ethically justified, the question as to what ethical obligations individuals have with regard to antibiotic consumption. I will address the first question in the next section (4), and the second question in Section 5.

## 4 SOLVING THE TRAGEDY OF THE COMMONS: INTERNALIZATION OF THE COSTS AND COMPENSATION FOR THE COSTS

In many cases, it is considered acceptable, if not ethically mandatory, to require those who, because of their behaviour, are responsible for a certain collective cost to compensate society for the cost they create or to do something that can prevent their behaviour from resulting in a net cost for society, that is, to internalize the cost. After all, bearing (at least part of) the costs entailed by one's behaviour is a significant part of what taking responsibility for one's behaviour entails.

Strictly speaking, internalization and compensation are different: internalization occurs when the benefit produced is commensurable with and equal to the cost. As put by Jonny Anomaly, internalizing a social cost requires ‘taxing negative externalities (…) at a rate that would offset the social cost of the activities that generate the externalities, and then (ideally) using the revenues from the tax to fund socially useful projects‘ (p. 433).24Anomaly, J. (2009). Harm to others: The social cost of antibiotics in agriculture. *Journal of Agricultural and Environmental Ethics*,* 22*, 423–435. Compensation implies instead that the cost cannot be offset because the benefit produced is not commensurable with the cost, but the individual responsible nonetheless makes up for it in some other way. Thus, someone might think that funding R&D and alternative conservation strategies would not count as internalization because they would not neutralize the cost that the individual is imposing with their current consumption of antibiotics. However, for the purpose of the present discussion, compensation and internalization can be seen as equivalent strategies, to the extent that they are both ways of contributing to conservation and innovation by those who have the responsibility to do so.

So, a first ethical solution to the tragedy of the commons represented by antibiotic consumption is that people who are responsible for the erosion of antibiotic effectiveness internalize the costs they are imposing on society, or anyway compensate society for the costs to which they are contributing. Taxation – in the form of a so‐called ‘Pigouvian tax’ – is well suited to addressing or preventing tragedy of the commons scenarios in general, and the tragedy of the commons of antibiotic consumption in particular,25Herrmann, M., & Ramanan L. (2010). Antibiotic effectiveness: New challenges in natural resource management. *The Annual Review of Resource Economics*,* 2*(4), 1–14; Littmann, J., & Viens, A. M. (2015). The ethical significance of antimicrobial resistance. *Public Health Ethics*,* 8*(3), 209–224, p. 214; Op. cit. note 2. because it would disincentivize consumption and could generate revenue that could be invested in conservation and innovation strategies.

The idea that a tax would be effective at reducing consumption is based on the standard assumption about behavioural economics that level of consumption is sensitive to price, which is one aspect of the more general ‘law of demand’ (Stigler 1947). The law of demand has some rare exceptions though. Some goods are not consumed less or are even consumed more as their price increases. In particular, people are prepared to spend money on certain ‘inferior’ goods – called ‘Giffen goods’ – when they are scarce, if necessary by foregoing other goods; for instance, they would often invest more on an ‘inferior good’ such as bread if its price increased, especially when the price of alternative goods (e.g. meat) increases as well.26Masuda, E., & Newman, P. (1981). Gray and Giffen goods. *The Economic Journal*,* 91*(364), 1011–1014. Another exception is the case of Veblen goods, that is, luxury goods such as diamonds, for which some people are willing to pay more because the higher the price, the higher the perceived benefits, for example in terms of social status (Malakhov 2012). Because consumption rate has sometimes been shown to be less sensitive to price when it comes to certain health goods,27Vuong, Q. H. et al. (2018). Healthcare consumers’ sensitivity to costs: A reflection on behavioural economics from an emerging market. *Palgrave Communications*,* 4*, 70. one might hypothesize that health goods such as antibiotics are also exceptions to the law of demand, perhaps a type of Giffen good. However, even conceding that this hypothesis is at least plausible, it would not be enough to undermine the effectiveness of the tax. As I said before, reducing consumption is only one way of promoting conservation: other conservation strategies, such as improving access to certain vaccines and developing new ones as well as improving hospital infection control,28Op. cit. note 17. would require investments of resources, especially in LMICs. For instance, ‘the introduction of the pneumococcal conjugate vaccine has reduced the burden of pneumococcal disease, avoided many prescriptions for antibiotics, and lowered rates of invasive disease caused by strains not susceptible to penicillin’.29Ibid, p. 1300. Promoting these alternative strategies requires investing resources, and taxing antibiotics in HICs would generate the necessary revenue. Furthermore, revenue from the tax could be used to fund innovation through R&D as well. Thus, even assuming that the antibiotic tax would not disincentivize consumption, it would contribute to internalizing or at least compensating for the costs of antibiotic consumption by contributing towards conservation and preservation strategies. Actually, the less effective the tax is at reducing consumption, the more revenue it would generate for alternative conservations strategies and for R&D, and vice versa.

A milder variant of an antibiotic tax could be a policy that sets a cap, say an annual upper limit, on the amount of tax‐free antibiotics that any doctor can prescribe to her patients. Above that limit, antibiotics could be heavily taxed. In this case, each doctor would probably be inclined to preserve their quota of tax‐free antibiotics for patients who need them the most, and therefore to prescribe antibiotics less often.

Yet another alternative taxation policy would be to tax only newer‐generation antibiotics, in order to preserve their effectiveness for as long as possible, in case the tax was effective at discouraging consumption.

Three aspects of the tax on antibiotics need to be specified. First, although I have said that my argument remains valid regardless of whether level of consumption will be sensitive to price, it is important to stress that there is scope for promoting such sensitivity: there will always be an amount of money that an individual would not be prepared to pay to access antibiotics, especially if the infection is mild and self‐limiting. So if the only aim was to reduce consumption, then it would just be a matter of finding the right amount that would sufficiently reduce consumption. Second, it is important to point out that a tax is applicable even in collectively subsidized healthcare systems where the cost of drugs is borne, at least in part, by the state. The cost of the drug is distinct from the tax: the former is what pharmaceutical companies are paid for selling the drug; the latter is what the state collects to fund conservation and innovation strategies. Thus the state can pay for the cost of the drug while levying a tax on consumers. Third, on any of the three variants of the taxation policies I have proposed, the tax should be on the patient, not on the doctor: as is the case with many other medical treatments, it is the patient's choice which treatment to receive, unless more restrictive policies than taxation – such as a ban on certain antibiotic use – are necessary (an issue I will touch upon in the final section). To the extent that the patient is responsible for the decision and the imposition of the cost, it is the patient who should be taxed when their best interest collides with the collective interest.

It is tempting to draw an analogy between a tax on antibiotic consumption and a tax on consumption of alcohol and tobacco, which also generates negative externalities, for instance in terms of expenditure to address the health consequences of their consumption. However, we need to be cautious in drawing such analogies. Obviously, a person can decide not to smoke or not to consume alcohol without significant costs to herself (actually, with significant benefits, although the addiction caused by alcohol and tobacco consumption does generate some individual costs to those who are forced to quit or reduce consumption). In contrast, foregoing antibiotics can entail significant costs to individuals in terms of deterioration of health due to infections, depending on the type of infection involved and on their general health status. Unlike the case of foregoing alcohol and tobacco, foregoing antibiotics can in some cases be considered supererogatory.

For the same reason, the justification for imposing a tax on antibiotics that are necessary to treat major and severe infections is weaker than it would be if foregoing antibiotics, i.e., avoiding paying the tax, were a costless option producing only minimal harms for the individuals (as is the case with alcohol and tobacco). Thus, while a tax on antibiotics needed to treat major infections, or infections in patients with compromised health status, would be very difficult to justify ethically, an ethical case could more easily be made for a tax on antibiotics used to treat minor or mild infections in otherwise healthy patients. In the next section, I will make my contribution to building such ethical case by arguing that foregoing antibiotics in certain cases is a moral obligation, and therefore a policy that restricts access to antibiotics in such cases has a strong ethical justification.

Granted, this type of policy would have two ethical costs: first, assuming that consumption rate is sensitive to price, some minor or mild self‐limiting infections might be left untreated, which, as some have argued, might be necessary in order to effectively contain ABR.30Foster, K., & Hajo, G. (2006). Do we need to put society first? The potential for tragedy in antimicrobial resistance. *PlosMedicine*,* 3*(2), 177–180.  Second, and related to this last point, such policies would probably result in inequalities in healthcare provision by leaving certain people, but not others, without effective antibiotic treatments: only those who are willing or can afford to pay the tax will have access to effective antibiotics.

The question is whether such costs are worth paying, and therefore whether the types of policies here suggested are ethically justified, all things considered. In the next two sections, I will suggest that they are.

## 5 AN ETHICALLY JUSTIFIED POLICY: TAXING ANTIBIOTICS FOR MILD AND SELF‐LIMITING INFECTIONS

As I have said above, the justification for taxing antibiotics is stronger when individuals have an independent moral obligation to forego antibiotics for the sake of the common good or when they have an independent moral obligation to internalize or compensate for the costs they are contributing to. The two moral obligations are related in the sense that the moral obligation to internalize or compensate for the cost of antibiotic consumption is the strongest when individuals are morally responsible for such costs, that is, when they fail to fulfil a moral obligation not to impose such costs or not to contribute (even probabilistically and/or imperceptibly, if there is a moral duty not to do so) to such costs in the first place. Of course, there might be other sources of the moral obligation to contribute to a common good in ways that are similar or equivalent to internalization or compensation; thus, also those who are not directly responsible for the erosion of antibiotic effectiveness might be ethically required to do something to address the problem (not least because each of us is a potential beneficiary of effective antibiotics). Here, I will focus on the moral obligations of those who are directly causally and morally responsible for ABR.

Now, individuals have a moral obligation to forego antibiotics when foregoing antibiotics would be, so to speak, a form of ‘easy rescue’,31Savulescu, J. (2007). Future people, involuntary medical treatment in pregnancy, and the duty of easy rescue. *Utilitas*,* 19*(1), 1–20. i.e., when the cost to them of foregoing antibiotics is sufficiently small. A duty of easy rescue is an intuitive and fundamental moral duty that all reasonable people can agree upon, regardless of the ethical theory or ethical viewpoint they adopt. For example, within Tim Scanlon's contractualist perspective, ‘[if] you can prevent something very bad from happening (…) by making only a slight (or even moderate) sacrifice, then it would be wrong not to do so’ (p. 224),32Scanlon, T. (1998). *What we owe to each other*. Cambridge, MA: Harvard University Press. and as Peter Singer famously put it, ‘if it is in our power to prevent something bad from happening, without thereby sacrificing anything of comparable moral importance, we ought, morally, to do it’.33Singer, P. (1972). Famine, affluence, and morality. *Philosophy and Public Affairs*,* 1*(3), 229–243, p. 230.


Note that Singer's formulation of the duty of easy rescue is more demanding than Scanlon's formulation: Singer leaves the possibility open that individuals ought to make great sacrifices when the harm prevented is comparatively great enough. In the case of antibiotic consumption, this type of moral duty might include, for example, the moral duty to leave serious infections untreated in order to preserve the common good of antibiotic effectiveness. However, as I suggested above, this kind of claim, and policies that would have such implications, are much more difficult to justify ethically. For this reason, I am not endorsing here the Singerian more demanding version of the duty of easy rescue (nor am I rejecting it; I want to remain neutral on the point). Rather, I will stick with Scanlon's version. The question I want to ask here is the following. What are the implications of this duty of easy rescue for the collective and individual moral obligation to reduce antibiotic consumption, and therefore for the justification of coercive policies that disincentivize, through taxation, the use of antibiotics?

Now, in certain circumstances, and specifically in the case of ‘milder and mostly self‐limiting bacterial infections’,34Op. cit. note 30, p. 178. such as for example respiratory tract infections in otherwise healthy patients, foregoing antibiotics for the sake of preserving the common good of antibiotic effectiveness does seem to be a form of easy rescue: the cost to individuals is sufficiently small, because we are talking of mild and self‐limiting infections, which would anyway be resolved without antibiotics in a slightly longer time; at the same time, the benefit to society in terms of preservation of antibiotic effectiveness is potentially very large, especially if a large enough population foregoes antibiotics for mild and self‐limiting infections.

This last consideration is worth examining as it requires us to clarify the concept of ‘rescue’ I have used so far. It is true that the moral responsibility to reduce the amount of antibiotics consumed (and the related responsibilities to internalize the costs of antibiotic consumption and to subsidize conservation and innovation strategies) is primarily a matter of collective, rather than individual, moral responsibility, in two senses. First, it is necessary that a sufficiently large portion of the population contributes to such enterprises in order for ABR to be significantly slowed down. Second, any individual's actual contribution to the collective cost might be negligible and in any case is only probabilistic; after all, any individual consuming antibiotics might not make a significant difference to the erosion of the common good because they might not pass their resistant bacteria on to other people. However, the fact that the obligation in question is primarily collective does not mean that individuals do not have a moral obligation to make their fair individual contribution, regardless of whether or not they do make a difference. The concept of ‘rescue’ here adopted should not be taken literally: the individual duty in question might not be a duty to directly benefit or prevent harm to another individual, as the literal meaning of ‘rescue’ suggests. The same duty justified by the small cost to the individual might arise from other ethical requirements, different from the requirement to benefit or to not harm others directly. As is the case with many other instances of collective obligations – think, for example, of the collective obligation to realize herd immunity through vaccinating a large enough portion of a given population – considerations of fairness in the distribution of certain burdens demand that each individual takes on herself her fair share of the burdens entailed by the collective obligation, at least as long as the burden is sufficiently small.35Giubilini, A., Douglas, T., & Savulescu, J. (2018). The moral obligation to be vaccinated: Utilitarianism, contractualism, and collective easy rescue. *Medicine Health Care and Philosophy*, *21*(4),547–560.


Thus, the justification for the tax and for the moral obligation to forego antibiotics in certain cases lies not only in the potential harm that any individual consumer of antibiotics poses on others, but also on the consideration that it is fair to contribute to the preservation of the common good, when it is sufficiently easy to do so. Thus, because there is a moral obligation to forego antibiotics in certain cases, the ethical justification for a policy that coerces individuals into foregoing antibiotics in such cases is very strong. Granted, the duty of fairness might well extend to cases in which making one's contribution is more burdensome; however, as I said, I will set aside these more controversial cases.

It is important to note that there are two distinct but complementary demands of fairness at stake here. If there is a collective obligation to reduce antibiotic consumption, each individual ought to do her fair share to reduce antibiotic consumption by forgoing some antibiotic use when doing so comes at a small individual cost. And if there is a collective obligation to internalize or compensate for the costs of antibiotic consumption, it is fair that those who are morally responsible for such costs – that is, those who have a moral obligation to forego antibiotics in certain cases, but fail to fulfil this moral obligation – primarily bear such costs, and therefore contribute to the internalization or compensation through taxation.

Indeed, foregoing or at least delaying antibiotics in the case of self‐limiting infections, such as respiratory tract infections in otherwise healthy patients, has already been proposed as a type of antibiotic stewardship program. Unfortunately, however, it is not a widely accepted practice: a study in 2011 found that the practice of ‘delayed prescribing’ in case of some respiratory tract infections – where foregoing antibiotics would be an ‘easy rescue’ – introduced in the U.K. by the National Institute for Health and Clinical Excellence was generally not adopted by healthcare practitioners.36Peters, S. et al. (2011). Managing self‐limiting respiratory tract infections: A qualitative study of the usefulness of the delayed prescribing strategy. *British Journal of General Practice*,* 61*(590), e579–e589. A taxed‐based policy that disincentivizes such uses of antibiotics might be effective in achieving the goals that this U.K. program failed to achieve, given certain behavioural economic assumptions. If it does not, it will nonetheless force those who are morally responsible for the erosion of the common good to internalize or compensate for the cost by funding conservation and innovation strategies.

Importantly, restrictive policies like the one I am defending might require adequate measures to ensure that the impact on those who decide to forego antibiotics because of the financial disincentive is minimized, and therefore that their sacrifice is actually a form of ‘easy’ rescue. Making the sacrifice as easy as possible is simply part of a state's obligation to ensure that the justification for its restrictive policies is the strongest possible from an ethical point of view. Such measures certainly include adequate medical follow‐up on the state of an individual's infection, but might also include some form of non‐financial compensation, for example prioritization on waiting lists for other types of health interventions, should the patient need them.

## 6 THE EQUALITY OBJECTION

Such a taxation policy might imply that some people will be coerced (to different degrees, depending on their financial circumstances) into foregoing antibiotics while other people will not, at least on certain behavioural economic assumptions. This raises ethical concerns about equality in access to healthcare. Simply put, the rich would be able to pay the tax and therefore to afford antibiotics for any infection, while the poor might have to leave certain mild and self‐limiting infections untreated for economic reasons. So far I have argued that the ethical case for a tax on certain uses of antibiotics is very strong; but is it strong enough to ethically justify such a policy, *all things considered*? Or would inequality in access to antibiotics represent a strong enough objection against the tax? Here, I am going to argue that it does not, given the constraints on the tax I have mentioned above.

Let me start by pointing out that the equality objection could be circumvented by simply banning the use of antibiotics for everyone in the circumstances I have described. This solution might at some point be necessary, if the taxation model does not prove effective. However, a ban is a measure of last resort and therefore it is not preferable to a milder policy such as taxation, which leaves at least some people the opportunity to treat their mild and self‐limiting infections. A principle of ‘least restrictive alternative’, widely appealed to in public health ethics,37Childress, J. F., Faden, R. R., Gaare, R. D., Gostin, L. O., Kahn, J., Bonnie, R. J., … Nieburg, P. (2002). Public health ethics: Mapping the terrain. *The Journal of Law, Medicine & Ethics: A Journal of the American Society of Law, Medicine & Ethics*,* 30*(2), 170–178, p. 173; Gostin, L. O. (2008). *Public health law: Power, duty, restraint*. (Revised and expanded 2nd ed.). London: University of California Press, p. 142. suggests that a policy A that is less restrictive than and at least as effective as a policy B ought to be preferred to policy B, *other things being equal*. Taxation, if effective at tackling ABR, is ethically preferable to a ban because it is less restrictive, that is, because it constrains individual liberties to a lesser degree and therefore allows at least some individuals to make a free choice whether or not to use antibiotics. One might reply here that, so to speak, things are not equal, because taxation has an equality cost that an outright ban does not have. But I have two responses to this reply.

First, a ban is preferable to a tax on grounds of equality only as a form of ‘levelling down’: we are making people worse off without making anyone better off than they would otherwise be, just for the sake of equality. But the idea that we would need to ‘level down’ in order to avoid inequalities is normally taken to be an objection to prioritizing equality, rather than a solution to the problem of equality. So it is not clear that a ban would be preferable to a tax just because it would fare better with regard to equality. Simply, the value of equality does not seem to outweigh the value of individual liberty or of expected utility when it requires to level down.

Second, and more importantly, how great a problem is inequality in the case here under scrutiny? There are two reasons for the view that unequal access to healthcare, and consequently an unequal distribution of health, is ethically problematic: first, with regard to the causes, unequal access to healthcare expresses an underlying injustice when we focus on its social determinants; second, with regard to the consequences, unequal access to healthcare might compromise something valuable, not only health itself and the related wellbeing, which are not positional goods, but also – as has been suggested – fair equality of opportunities.38Daniels, N. (2008). *Just health*. Cambridge: Cambridge University Press. In other words, those who receive worse (or less) healthcare might be further penalized with respect to very valuable things, apart from suboptimal health; examples offered have included unequal risks of stigmatization and of unemployment and unequal chances to enjoy a certain amount of pension years.39Haverkamp, B. et al. (2018). Why socio‐economic inequalities in health threaten relational justice. A proposal for an instrumental evaluation. *Public Health Ethics*, *11*(3), 311–324. https://doi.org/10.1093/phe/phy020. However, while it is true that the social determinants of health often reflect social inequalities that need to be addressed – an issue that is beyond the scope of this paper – it does not seem that foregoing antibiotics in the circumstances I have described would have such dramatic consequences both for health as valuable in itself (quite apart from equality concerns) and for health as instrumentally valuable for equality purposes, given that the infections in question are short‐lived in otherwise healthy patients. In other words, to use the terminology of the capability approach that grounds one of the most authoritative accounts of (a certain form of) egalitarianism,40Anderson, E. (1999). What's the point of equality? *Ethics, 109*, 287–337. foregoing antibiotics in the circumstances I have described would not deprive individuals ‘of the capabilities needed to function as equals’ (p. 313),41Op. cit. note 39. at least not for a significantly long time. Thus, it does not seem that leaving mild and self‐limiting infections untreated in otherwise healthy patients would significantly compromise fair equality of opportunities. Inequality might be a serious ethical concern (among others) only if antibiotics were taxed in the case of more severe infections.

With the kind of taxation policy I am proposing, there would still be *some* inequalities in levels of wellbeing experienced, for sure, and I am not denying that this is an ethical cost. However, given how vital it is to contain ABR urgently, it is an ethical cost worth paying, because those who would be worse off as a result of the unequal access to antibiotics would still be well off *enough* – given that they would still have tax‐free access to antibiotics used to treat more serious infections and that they might be compensated. They would still be well off enough both in absolute terms and with regard to those aspects that make unequal access to healthcare ethically unacceptable. In other words, the policy I am proposing would sacrifice a strictly egalitarian ideal but would preserve a sufficientarian conception of distributive justice, which seems an acceptable compromise between the values of expected utility (in terms of containment of ABR) and equality. Equality is an important value, but in certain circumstances it cannot be the most important value.

Some have suggested that a fixed‐amount modest surcharge on antibiotics42Battin, M. et al. (2009). *The patient as victim and vector: Ethics and infectious disease*. Oxford: Oxford University Press. would be preferable to a tax because the surcharge would not be calculated on the basis of the actual cost of antibiotic consumption and therefore could be kept sufficiently low so as not to price poor people out of the market.43Op. cit. note 19. However, if I am right that inequality in access to healthcare is not a very strong objection in the case of a tax like the one I am proposing, then such a tax is preferable to a fixed‐amount small surcharge because a small surcharge without an equality cost would certainly not be effective at disincentivizing consumption (if it was, it would not have that kind of equality cost) and might not be sufficient to adequately fund conservation and innovation strategies. A tax could be effective for either or both purposes, once the appropriate amount to be levied was found.

## 7 CONCLUSION

I have argued that, because individuals have a moral obligation to forego antibiotics in the case of mild and self‐limiting infections (and absent any other relevant health issue), there is a very strong ethical case for a tax on antibiotics that disincentivizes individuals from accessing antibiotics to treat minor and self‐limiting infections or that forces individuals to take responsibility for failing to do so by internalizing or compensating for the cost. Such a policy would simply force individuals to fulfil a moral obligation that they anyway have, and the ethical cost in terms of unequal access to healthcare would be small enough to make such a policy ethically permissible all things considered. What exact form such a taxation policy should take requires a separate discussion. Whether some taxation policy would sufficiently slow down ABR and generate enough revenue to allow funding of conservation and innovation strategies is an empirical issue that would need to be tested. Here, my aim was only to provide an ethical analysis and an ethical justification for this type of policy. More coercive policies or policies that demand greater individual sacrifices might be necessary, but they would require a stronger ethical justification.

## ACKNOWLEDGMENTS

Work on this article was funded by the Oxford Martin School (Program: Collective Responsibility for Infectious Disease) and by the Wellcome Centre for Ethics and Humanities, University of Oxford, which is supported by a Wellcome Centre Grant (203132/Z/16/Z). The article is made Open Access thanks to the Wellcome Trust.

## CONFLICT OF INTEREST

The author declares no conflict of interest.

